# Emotional regulation, empathy and surgical performance inundergraduate dental students: A prospective cohort study

**DOI:** 10.4317/medoral.27960

**Published:** 2026-03-07

**Authors:** Alba Ballestero-Pérez, Idana Martínez-Mayoral, Cristina de-la-Rosa-Gay, Marta García-García, Mari Aguilera, Rui Figueiredo, Eduard Valmaseda-Castellón, Alba Sánchez-Torres

**Affiliations:** 1 Faculty of Medicine and Health Sciences, University of Barcelona, Barcelona, Spain; 2 IDIBELL Research Institute, Barcelona, Spain; 3 Faculty of Psychology, University of Barcelona, Spain; 4 Neuroscience Institute (UBNeuro), Universitat de Barcelona, Barcelona, Spain

## Abstract

**Background:**

Dental students experience high stress from academic and clinical demands, which may affect performance and well-being. Surgical procedures often increase patient anxiety, adding complexity for students. This study explored the relationship between perceived difficulty, emotional regulation, and empathy, and their impact on clinical outcomes and patient satisfaction.

**Material and Methods:**

A prospective cohort study included fourth-year dental students in the Clinical Oral Surgery and Implantology subject at the University of Barcelona Dental Hospital. Data collected comprised demographics, emotional regulation difficulties, and preoperative anxiety, using validated questionnaires. Procedure-related variables (perceived difficulty, operative time, complications) and patient data (anxiety, perceived empathy, satisfaction) were recorded.

**Results:**

Thirty-six students performed 108 extractions on 72 patients, with a mean operative time of 38.2±19.9 minutes. Emotional regulation difficulties were not associated with surgical difficulty, empathy, operative time, or complications. Interestingly, patient state anxiety was significantly related to higher difficulty on emotional regulation by the undergraduate. No significant correlation was found between patient anxiety and number of visits. Perceived empathy positively correlated to patient satisfaction.

**Conclusions:**

Empathy and emotional regulation are essential for enhancing patient experience and outcomes. Our findings suggest that students' emotional regulation difficulties are related to situational anxiety during clinical encounters. These results support integrating interpersonal skills into dental education to improve clinical practice and patient-centered care.

## Introduction

Undergraduate students enrolled in professional degree programs are susceptible to psychological issues caused by stressors such as the responsibility demanded by their training. In the health sciences field, some of these stressors may potentially cause harm or pain to patients (1 , 2). Stress is defined as "the nonspecific response of the body to any demand" and involves physical and emotional reactions. Factors such as a tendency toward perfectionism, a heavy workload, fear of failure, and lack of free time have been identified as causes of sustained stress in students. While acute stress triggers a physiological coping response and mild stress may have beneficial effects on memory and cognition, prolonged chronic stress can lead to symptoms of anxiety, burnout, physical health issues, and even depression (3).

Dental students are required to develop both technical and non-technical skills, including interpersonal abilities (1 , 2). They are immersed in a learning curve that involves developing autonomy in clinical skills. In this process, a reflective practice and detailed feedback from a mentor or instructor help students identify their abilities and learning needs (4). Likewise, promoting self-assessment facilitates the recognition of areas for improvement and fosters a commitment to lifelong learning (5).

It is worth noting that medical procedures can provoke anxiety, as well as psychological and physiological distress, in patients, both adults and children (6). In particular, anxiety or dental fear may lead to the avoidance of dental visits and a worsening of oral health (7). Moreover, anxiety exacerbates perceived pain by modulating neural activity, thereby increasing the need for sedation and analgesia (6 - 8). The global prevalence of dental anxiety and fear among patients ranges from 6-21% (9). Specifically, oral surgery procedures may trigger a high level of anxiety due to the administration of local anesthesia, the noise and vibration of rotary instruments, the force needed to extract teeth, and the occurrence of postoperative complications (10). Communication and the patient-clinician relationship are key elements in such procedures. Therefore, the use of specific tools to assess dental anxiety, such as the Modified Dental Anxiety Scale (MDAS), might be useful in clinical studies (10).

The emotional regulation strategies of dental students may play a key role in improving their concentration, communication skills, and manual dexterity when performing procedures. Similarly, the extrinsic emotional regulation of patients can have beneficial effects on their own intrinsic emotional state during treatment (11). In the surgical setting, certain stressors, such as distractions and time pressure, have been identified (12). In outpatient dentoalveolar surgeries, patient behavior can increase perceived stress and/or workload in students during their training phase. This could negatively affect their performance, increase the duration of the procedure or the risk of complications (13). Although several studies address emotional regulation and stress, few have explored how such regulation impacts students during their initial clinical experiences with patients, as well as its potential influence on the empathy perceived by the latter.

The primary aim of the present study was to assess the relationship between the perceived difficulty of dental extractions, the emotional regulation difficulties of the students, and the degree of empathy perceived by the patients. The secondary objectives were to evaluate the surgical performance, based on operative time and the occurrence of intraoperative complications, and its relationship with the emotional regulation difficulties of the students; and to correlate the number of appointments, the level of anxiety, and the overall patient satisfaction.

The main hypothesis of the study was that students with higher emotional regulation difficulty scores perceive the procedures as more challenging, and their patients report lower perceived empathy.

## Material and Methods

The present prospective cohort study included 36 students from two clinical practice groups enrolled in the Clinical Oral Surgery and Implantology subject (31.3% of the total cohort; n=115), taught in the fourth year of the Dentistry degree at the University of Barcelona. The study was performed between December 2024 and May 2025. The manuscript was written in accordance with the STROBE (Strengthening the Reporting of Observational studies in Epidemiology) guidelines (14).

The study protocol (reference 48/2024) was approved by the Ethics Committee of the University of Barcelona Dental Hospital (CEIm Hospital Odontológic Universitat de Barcelona), and the study was conducted in accordance with the Declaration of Helsinki on research involving human subjects (15). All students and patients agreed to participate in the study and signed an informed consent form. They were also provided with an information sheet detailing the study's procedures and objectives.

Eligibility criteria

Students enrolled in the Clinical Oral Surgery and Implantology subject with patients undergoing dental extractions were included. These students were in their first year of clinical practice, having previously completed preclinical training on dental phantom models. During the procedures, students were closely supervised by assistant professors, with a ratio of 2 professors per 5 clinical units.

Patients attending a first-time consultation or a postoperative follow-up appointment were excluded.

Study variables and procedure

Initially, the students were required to complete a data form including gender, age, prior academic degrees, frequency of physical exercise, tobacco and alcohol consumption, average hours of sleep per day, and the number of dental extractions performed up to that point. In addition, they were asked to complete two questionnaires: The Difficulties in Emotional Regulation Scale (DERS) (16) and the State-Trait Anxiety Inventory (STAI-S and STAI-T) (17). Simultaneously, patients were asked to fill out a form including questions about gender, age, number of previous appointments with the same student, whether it was their first time undergoing the procedure, and any previous negative experiences at the dentist. If such experiences had occurred, they were asked to select the option that best described the cause: Lack or excess of information about the procedure, the administration of local anesthesia, the sensation of having a numb mouth/area, the needle, physical sensations (such as vibration, pressure, tension), the sound and/or smells, lack of empathy and communication with the attending dentist, experiencing unpleasant physical reactions (distress, rapid heartbeat, dizziness), overall discomfort or fear without being able to specify the cause, or the symptoms experienced after the visit (pain, swelling, impact on social life). In addition, the patients were asked to complete the MDAS questionnaire (10).

**Figure 1:**
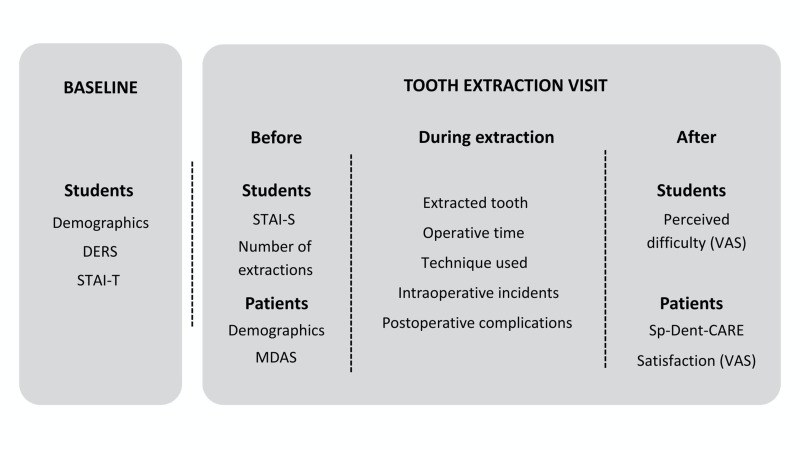
Timeline of the study. DERS: Difficulties in emotional regulation scale. STAI-T: State-Trait anxiety inventory. MDAS: Modified dental anxiety scale. VAS: Visual analogue scale.

After the procedure, the student's perceived difficulty with the extraction was recorded based on a visual analogue scale (VAS) from 0 to 10cm, where 0: "not difficult at all" and 10: "most difficult". Likewise, the patients completed the Sp-Dent-CARE questionnaire (18) and rated their perceived satisfaction with the student using another VAS (Figure 1).

1

During the procedure, the following clinical variables were recorded: Extracted tooth, operative time from local anesthesia to patient discharge, surgical technique (need of a flap elevation, bone removal or tooth sectioning), and the occurrence of intraoperative incidents (root fracture, fracture or displacement of the buccal and/or lingual cortical plate, or damage to the adjacent teeth). Immediate postoperative complications were also documented, including bleeding, arterial hypotension or vasovagal reaction, nausea or vomiting, and other adverse reactions.

If needed, closure was performed using resorbable sutures (polyglycolic acid, PGA 4/0; Laboratorios Aragó, Barcelona, Spain), and the wound was compressed using gauze until bleeding stopped.

Statistical analysis

A descriptive and bivariate analysis of the collected data was conducted. The normality of the study variables was verified using the Shapiro-Wilk test. The relationship between emotional regulation difficulty and operative time, perceived procedure difficulty, patient-perceived empathy, and patient satisfaction with the appointment was assessed using Pearson or Spearman correlations with the Bonferroni correction. Additionally, the relationship between the number of appointments, the degree of perceived empathy, and the patient's overall satisfaction with the visit was also explored. In cases where the assumption of normality was not met, non-parametric tests were used. Finally, a multivariate linear regression model for emotional regulation difficulty was performed. Assessment of interactions and possible confounding factors helped select the most parsimonious model. Statistical significance was considered for p&lt;0.05. The STATA 15.1 statistical package (StataCorp®, College Station, TX, USA) was employed for the analysis.

## Results

A total of 72 patients, 36 men (50%) and 36 (50%) women, with a mean age of 55.3 years (standard deviation (SD)=19.5) were treated by 36 students (32 women (89.9%) and 4 men (11.1%), with a mean age of 24.5 years (SD=4.4).

A total of 108 dental extractions were performed, involving 94 conventional and 14 surgical procedures, with a mean duration of 37.7 minutes (SD=19.6). The mean perceived difficulty was 48.1 mm (SD=26.7), assessed on a 100mm VAS.

The most frequent intraoperative incident was root fracture (n=9, 8.3%), followed by flap tear (n=1, 0.9%), luxation of the adjacent tooth (n=1, 0.9%), and bleeding (n=1, 0.9%). Fractured teeth were removed, and the flap tear was sutured. Bleeding was managed by compression with a gauze soaked in tranexamic acid (Amchafibrin, Meda Pharma SL, Madrid, Spain). No other complications were reported. Regarding immediate postoperative complications, there was one case of bleeding and one case of arterial hypotension (1.4 %).

Table 1 presents the main characteristics of the participating students. Nearly 90% were women, and around 40% were dental hygienists. The perceived difficulty following tooth extractions according to the VAS indicated a moderate level of complexity.

Table 1Table 2 shows the demographic characteristics of the patients treated, as well as their level of anxiety, satisfaction, and perceived empathy. Nearly half of the patients (n=35, 48.6%) had attended two or more appointments with the same student, and most had undergone previous dental extractions (n=62, 86.1%). More than one-third of the participants (n=26, 36.1%) reported previous negative experiences in the dental setting. The most frequently reported reasons for dental anxiety were "lack of empathy and communication with the attending professional and/or team" (n=9, 12.5%), "the entire situation made me uncomfortable or fearful, without being able to specify a particular cause" (n=8, 11.1%), and "the symptoms experienced after the visit" (n=8, 11.1%).

Table 2The mean MDAS score (7.8, SD=3.2) indicated low to moderate anxiety. The mean overall satisfaction was high (92.8, SD=16.8). Finally, the mean Sp-Dent-CARE scale score was 46.3 (SD=6.4), indicating a high perceived empathy. No gender-related differences (p=0.129) were observed.

No associations were found between negative past experiences and empathy (p=0.498) or dental anxiety according to MDAS (p=0.143) or STAI-S (p=0.556).

The variables did not follow a normal distribution, so Spearman correlations were performed. The correlation matrix (Table 3) shows no relationship between the emotional regulation of the students, the perceived surgical difficulty, and the level of empathy perceived by the patient. Operative time was significantly correlated to perceived surgical difficulty.

Table 3Patients who reported higher empathy from the students were significantly more satisfied with the appointment (r=0.339; p=0.004). Patient anxiety was not related to the number of appointments with the same student (p=0.070), the level of perceived empathy (r=-0.033; p=1.000), or overall satisfaction with the visit (r= -0.122; p=0.920).

Finally, the multivariate linear regression model (Table 4) showed a significant linear correlation between patient state anxiety and emotional regulation difficulties of the undergraduate students (p=0.036). No significant correlations were found for gender and age of patients, anxiety according to MDAS scale, total number of tooth extractions performed by the student and the surgical technique.

Table 4

## Discussion

The main results of the present study show no correlation between the perceived difficulty of the procedure, the students' emotional regulation, and the empathy perceived by the patients. These results suggest that perceived surgical difficulty is more closely related to technical factors than to the student's emotional abilities. Furthermore, the incidence of complications was low, suggesting that most procedures were done correctly. Likewise, the fact that empathy perceived by the patient did not vary according to the student's emotional regulation could indicate that patients are not fully sensitive to the operator's internal states. Other aspects, like the communication abilities, might have a higher impact for the patients. It is also important to stress that the multivariate analysis indicated that students' emotional regulation difficulties are associated with the patients' anxiety during the clinical appointment rather than with surgical technique or patient characteristics.

The majority of students were women, had prior training in dental hygiene, and maintained healthy lifestyle habits. Moreover, they presented low to moderate preoperative anxiety.

Surgical procedures perceived as being more complex required longer operative times. However, no relationship was found between difficulties in emotional regulation and either increased operative time or the occurrence of complications. Thus, these results indicate that emotional regulation does not appear to have a direct impact on performance (such as the duration or safety of the procedure) or on interpersonal variables (such as empathy perceived by the patient). It should be noted that these results occur in a supervised clinical practice environment. This means that other variables such as faculty guidance, standardized protocols, or the specific technique of the task may influence students' emotional competencies and patient experience.

The surgical setting is highly demanding, and the integration of mental skills and emotional self-regulation are key components for optimizing both clinical performance and the operator's experience during the procedure (19). In fact, the ability to regulate emotion is a core component of emotional intelligence and has been identified as an effective method for reducing stress in healthcare settings. In studies conducted among dental students, higher emotional intelligence has been associated with better problem-solving, decision-making and effective communication. Replacing negative emotions with more adaptive ones, such as confidence and empathy, is crucial to individual success and development (20 , 21). In this context, the ability to identify, understand, and regulate one's own emotions, as well as those of others, has been linked to a significant reduction in stress and a more positive attitude towards the profession (20).

Such emotional management is particularly relevant for healthcare professionals, including dental students, as poor regulation may lead to deterioration of mental health (22). The results of the present study indicate that the evaluated students had few difficulties in emotional regulation.

The dentist-patient relationship is essential for creating an atmosphere of trust, particularly in patients who experience fear or anxiety regarding dental treatment. Clear communication enhances patient satisfaction, adherence to professional recommendations, and the perceived quality of care. In this context, the dentist's empathy is a key factor in reducing fear and increasing perceived satisfaction (23). For this reason, the rubric we routinely use to evaluate students in the clinical oral surgery and implantology subject (PIETA rubric for Punctuality, Interest, Empathy, Technique, Autonomy) (24), explicitly includes empathy as a core dimension, reflecting its fundamental role in the dentist-patient interaction.

In the present study, a relationship between the levels of empathy and a higher overall satisfaction with the appointment was observed. Likewise, Corah et al. (25) found that qualities such as kindness, empathy, and the general demeanor of the dentist positively contribute to patient satisfaction and dental anxiety reduction. Similarly, Jones et al. (26) identified a relationship between patient satisfaction and a reduction in anxiety.

Trust is a key skill in establishing an effective dentist-patient relationship, as it promotes adherence to treatment and may reduce patient anxiety (27). Thus, empathy is considered a relevant component of clinical competence and significantly contributes to improved healthcare outcomes (28). It is also worth noting that this may have positive implications for professionals, as it can lead to greater job satisfaction and lower levels of stress (29).

The low incidence of complications could be due to the preclinical training performed in the previous semester. Students first practice tooth extractions on models, including conventional procedures as well as simulated bone removal and tooth sectioning. Also, the present sample demonstrated a high level of empathy, which is a positive aspect in their professional development. The Hawthorne effect could not be ruled out, as students knew that patients would rate perceived empathy. In fact, our students are routinely assessed using the PIETA rubric (Punctuality, Interest, Empathy, Technique, Autonomy) (24), which includes empathy towards the patient as a dimension.

A study by Smith et al. (29) reported higher levels of empathetic traits among female dentists. We were unable to evaluate this aspect since our sample only included four men.

One of the main limitations of this study is the direct supervision by faculty staff. Students have very limited clinical experience and often require help during the procedures. Another drawback of the current research is the limited number of extractions performed by each student, which could lead to differences in their learning process and could also limit the statistical power. Although it was explored as a covariate in the multivariate analysis, the relatively homogeneous experience of students could impair detection of small or moderate effects. Therefore, the absence of significant associations should be interpreted with caution.

This study provides relevant evidence on the role of emotional and relational skills in the clinical training of undergraduate dental students. A strong correlation was observed between the perceived empathy and the overall satisfaction of patients, which highlights the need to integrate communication skills into the clinical curriculum. In addition, clinical teachers, beyond technical supervision, could also benefit from specific training aimed at providing feedback, emotional support and the teaching of non-technical skills.

Our study did not include longitudinal follow-up of emotional competencies, nor measurable behavioral variables such as verbal and non-verbal communication, which may influence patient perception. These aspects represent limitations, and future research should incorporate longitudinal designs and observational measures. Future studies should also consider using structured frameworks such as the NOTSS (Non-Technical Skills for Surgeons) system (30) to improve the assessment of competencies, such as decision-making, teamwork, clinical communication and emotional management during undergraduate oral surgery training. Furthermore, educational interventions to acquire emotional regulation strategies should be implemented, especially in challenging clinical situations that require managing patients with dental anxiety.

## Conclusions

Difficulties in emotional regulation of the students did not influence the perceived difficulty of the procedure, the operative time, the occurrence of complications or the empathy perceived by the patients. Our findings suggest that students' emotional regulation difficulties are related to anxiety during clinical appointments. However, these findings should be interpreted within the context of a highly supervised undergraduate clinical environment, where continuous faculty guidance and structured clinical protocols may mitigate the potential impact of individual emotional regulation on clinical performance and patient perception.

Similarly, the number of previous appointments with the same student was not significantly associated with patient anxiety levels. However, the perceived empathy was significantly correlated to patient satisfaction. The findings underscore how interpersonal skills, such as empathy and emotional regulation, contribute to better patient experiences and outcomes, and highlight the need to integrate these competencies into clinical dental training programs.

## Figures and Tables

**Table 1:** Table Main characteristics of the students included in the study.

Student variables		Mean (SD)/N (%)
Age		24.6 (4.4)
Gender	Male	4 (11.1%)
	Female	32 (88.9%)
Previous degree	None	13 (36.1%)
	Dental hygienist	15 (41.7%)
	Dental technician	1 (2.8%)
	Other	7 (19.4%)
Physical activity (times/week)		2.4±1.5
Sleep (hours/day)		6.7±0.8
Smoking habit	Non-smoker	30 (83.3%)
	Smoker	6 (16.7%)
	Former smoker	0 (0%)
Alcohol consumption	Never	13 (36.1%)
	Occasional	23 (63.9%)
	Daily	0
Difficulties in Emotion Regulation Scale	Non-acceptance of emotional responses	13.6 (±6.8)
	Difficulty engaging in goal-directed behavior	9.8 (±3.7)
	Impulse control difficulties	8.6 (±4.6)
	Emotional awareness	8.7 (±3.3)
	Limited access to emotion regulation strategies	8.1 (±4.4)
	Emotional clarity	8.2 (±2.6)
	Total score	56.3 (±21.3)
Trait-Anxiety (STAI-T)		25.4 (±3.7)
State-Anxiety (STAI-S)		25.5 (±3.2)

SD: Standard deviation. N: Number. STAI: State-Trait Anxiety Inventory. VAS: Visual analogue scale.

**Table 2:** Table Characteristics of the patients included in the study.

Patient variables		Mean (SD) / N (%)	
Age		55.3 (19.5)	
Gender	Male	36 (50.0%)	
	Female	36 (50.0%)	
Number of appointments with the same student	First time	20 (27.8%)	
	Second visit	17 (23.6%)	
	2 or more visits	35 (48.6%)	
First time undergoing the procedure	Yes	10 (13.9%)	
	No	62 (86.1%)	
Previous negative experiences	Yes	26 (36.1%)	
	No	46 (63.9%)	
Reason	Lack/excess of information	Yes	7 (9.7%)
		No	65 (90.3%)
	Administration of anesthesia	Yes	3 (4.2%)
		No	69 (95.8%)
	Sensation of numbness in the mouth	Yes	6 (8.3%)
		No	66 (91.7%)
	Needle	Yes	5 (6.9%)
		No	67 (93.1%)
	Physical sensations	Yes	5 (6.9%)
		No	67 (93.1%)
	Sounds and/or smells	Yes	4 (5.6%)
		No	68 (94.4%)
	Lack of empathy and communication	Yes	9 (12.5%)
		No	63 (87.5%)
	Unpleasant physical reactions	Yes	7 (9.7%)
		No	65 (90.3%)
	The entire situation was distressing	Yes	8 (11.1%)
		No	64 (88.9%)
	Symptoms after the visit	Yes	8 (11.1%)
		No	64 (88.9%)
Degree of dental anxiety (MDAS)		7.8 (±3.2)	
Satisfaction with the visit (VAS)		92.8 (±16.8)	
Perceived empathy (Sp-Dent-CARE)		46.3 (±­6.4)	

MDAS: Modified Dental Anxiety Scale. VAS: Visual analogue scale. Sp-Dent-Care: Interpersonal empathy in dental care.

**Table 3:** Table Correlation matrix of difficulties in emotional regulation, perceived difficulty, perceived empathy and operative time (n=72).

	DERS Scale	Perceived difficulty	Perceived empathy	Operative time
DERS Scale	-			
Perceived difficulty	r = -0.071	-		
Perceived empathy	r = 0.151	r = -0.209	-	
Operative time	r = -0.073	r = 0.516***	r = -0.179	-

DERS: Difficulties in Emotional Regulation Scale. ***p<0.001

**Table 4:** Table Multivariate linear regression model for emotional regulation difficulty measured through DERS scale.

	Coefficient	SE	95% CI	pvalue
Gender (patient)	2.12	3.99	-5.84 to 10.08	0.596
Age (patient)	-0.89	0.11	-0.17 to 0.25	0.706
Number of extractions	0.04	0.87	-2.63 to 0.84	0.306
MDAS	-0.27	0.63	-1.52 to 0.99	0.676
State-Anxiety	1.18	0.55	0.08 to 2.29	0.036*
Surgical technique	0.61	5.50	-10.38 to 11.61	0.911

DERS: Difficulties in Emotional Regulation Scale. MDAS: Modified Dental Anxiety Scale. SE: standard error. CI: confidence interval. *Significant value at p<0.05.

## Data Availability

Declared none.
